# Supplementary material for: Blood-based quantification of Aβ oligomers indicates impaired clearance from brain in ApoE ε4 positive subjects
Source: Commun Med (Lond). 2024 Dec 10;4:262. doi: 10.1038/s43856-024-00690-w (PMC11631981; doi:10.1038/s43856-024-00690-w)
Supplement: Supplementary file 2 — Supplementary Information [file 43856_2024_690_MOESM2_ESM.pdf]

Blood-based quantification of Aβ oligomers indicates impaired clearance from brain in ApoE ε4 positive subjects

Lara Blömeke, Fabian Rehn, Marlene Pils, Victoria Kraemer-Schulien, Anneliese Cousin, Janine Kutzsche, Tuyen Bujnicki, Silka D. Freiesleben, Luisa-Sophie Schneider, Lukas Preis, Josef Priller, Eike J. Spruth, Slawek Altenstein, Anja Schneider, Klaus Fliessbach, Jens Wiltfang, Niels Hansen, Ayda Rostamzadeh, Emrah Düzel, Wenzel Glanz, Enise I. Incesoy, Katharina Buerger, Daniel Janowitz, Michael Ewers, Robert Perneczky, Boris-Stephan Rauchmann, Stefan Teipel, Ingo Kilimann, Christoph Laske, Matthias H. Munk, Annika Spottke, Nina Roy, Michael T. Heneka, Frederic Brosseron, Michael Wagner, Sandra Roeske, Alfredo Ramirez, Matthias Schmid, Frank Jessen, Oliver Bannach, Oliver Peters, Dieter Willbold

Supplementary Figures and Tables

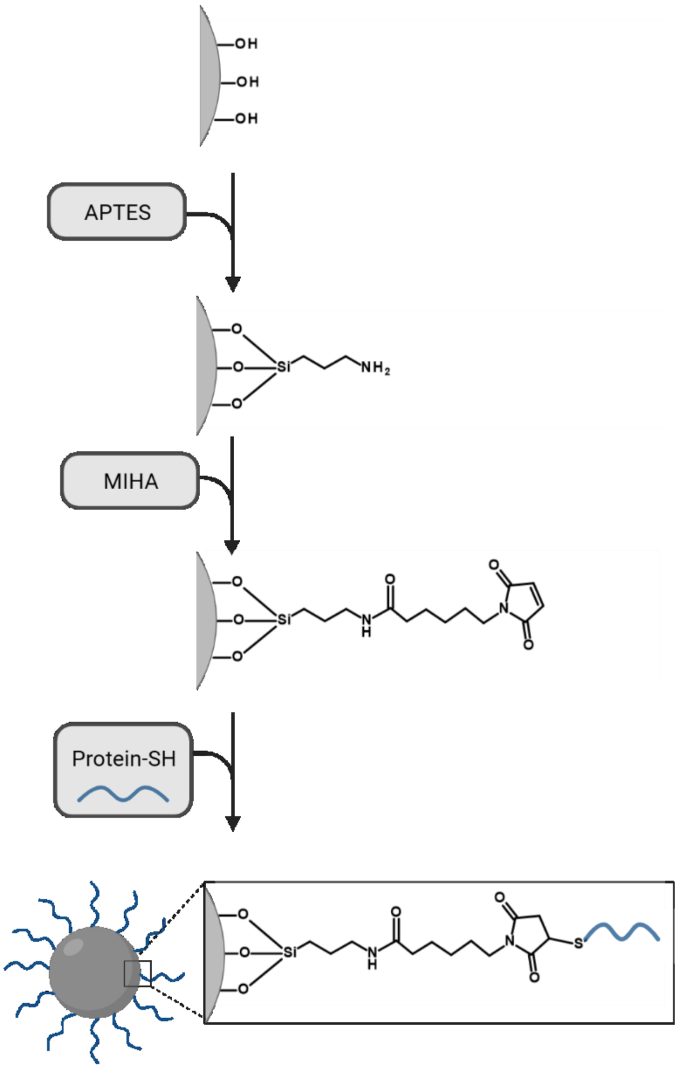

Supplementary Fig. 1 Synthesis of protein conjugated SiNaPs.

The process of protein conjugation of SiNaPs consists of three main steps. First, the silica core is functionalized with APTES. As a second step, MIHA is added as a crosslinker between the SiNaPs core and the protein. The use of maleimide as functional group in combination with the reaction conditions allows a directed coupling with the thiol group at the modified C-terminus of the protein. The resulting protein conjugated SiNaPs imitate a protein aggregate with multiple binding sites for the antibody but showing a unique size distribution, high stability, and defined epitope number [1].

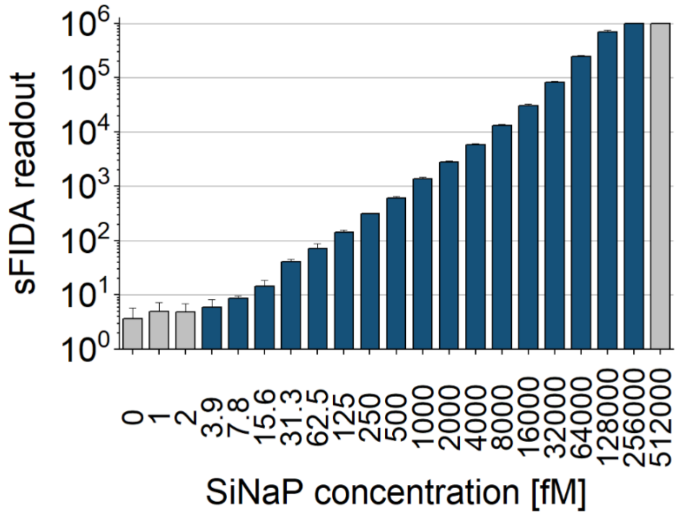

Supplementary Fig. 2 Dynamic range of SiNaP calibration

In an independent experiment, the dynamic range of SiNaP calibration standard spiked in plasma was determined. Two-fold serial dilutions were performed starting at 512 pM down to 1 fM. ULOQ was determined at 256 pM. The average dilution linearity between 3.9 fM and 256 pM was 0.91. The standard deviation was calculated across the four replicates. Please, note the logarithmic scale.

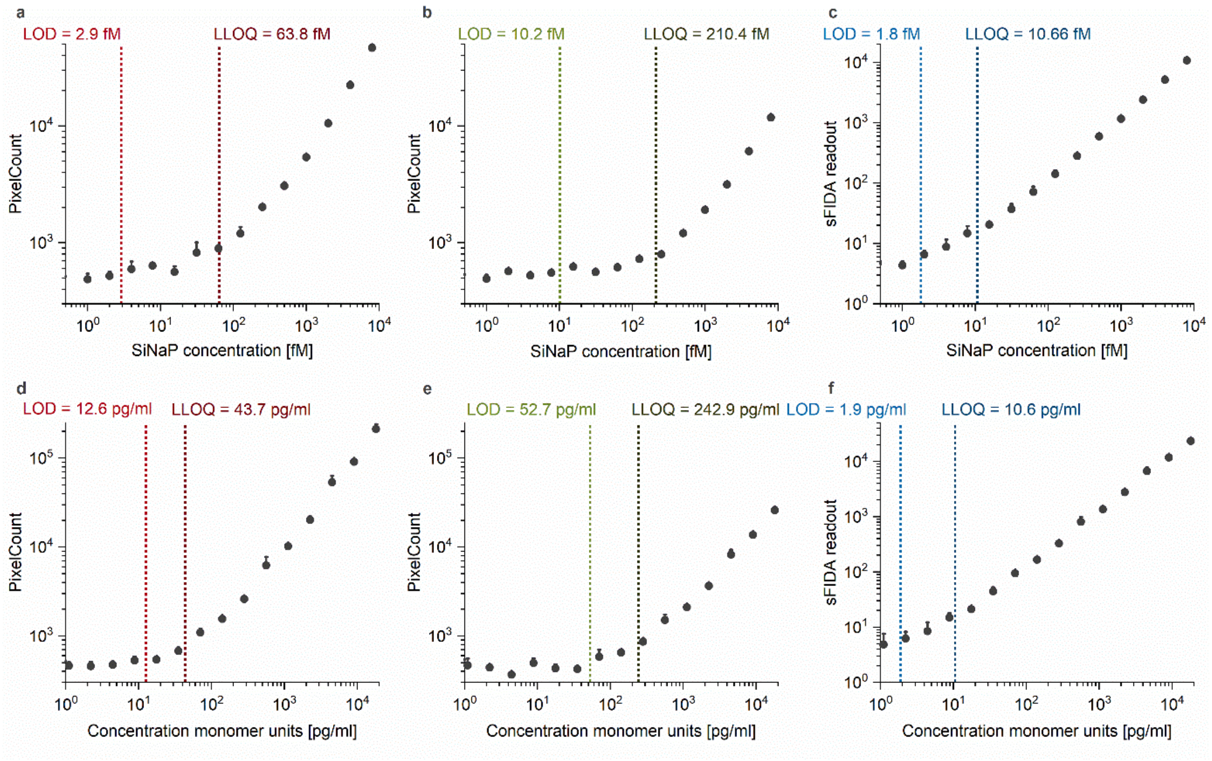

Supplementary Fig. 3 Linear dilution of Aβ$_{1-15}$ SiNaPs and Aβ$_{1-42}$ aggregates spiked in plasma. Shown are dilution series for Aβ$_{1-15}$ SiNaPs for the red (CF 633) **a** and green (CF488) **b** fluorescence channels, and the colocalization **c** and the corresponding limit of detection (LOD) and lower limit of quantification (LLOQ). Similarly, dilution series of synthetic Aβ$_{1-42}$ aggregates for the red (CF633) **d** and green (CF488) **e** fluorescence channels and colocalization **f** with calculated LOD and LLOQs are shown. The standard deviation was calculated across the four replicates. Please, note the logarithmic scale.

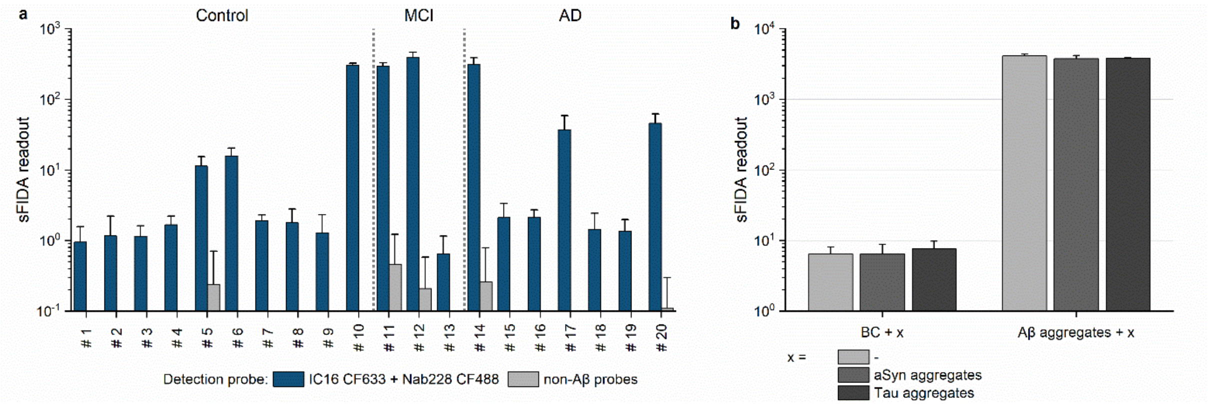

Supplementary Fig. 4 Assay controls with non-Aβ probes (a) and αSyn and Tau aggregates.

a sFIDA readouts of the samples (#1 to #20) of the validation cohort for detection with anti-Aβ detection antibodies IC16 CF633 in combination with Nab228 CF488 (blue) and non-Aβ probes MOPC-21 CF633 in combination with 211 CF488 (grey). While the samples detected with anti-Aβ antibodies showed an sFIDA readout ranging from approximately 1 pixel to 500 pixels, the samples did not give a signal when detected with antibodies who do not recognize Aβ. **b** Neat plasma and 1 nM (18 ng/ml) Aβ aggregates (concentration based on the monomer unit concentration) were spiked with 1 nM of αSyn and Tau aggregates, respectively. Standard deviation was calculated across the four replicates. Please, note the logarithmic scaling.

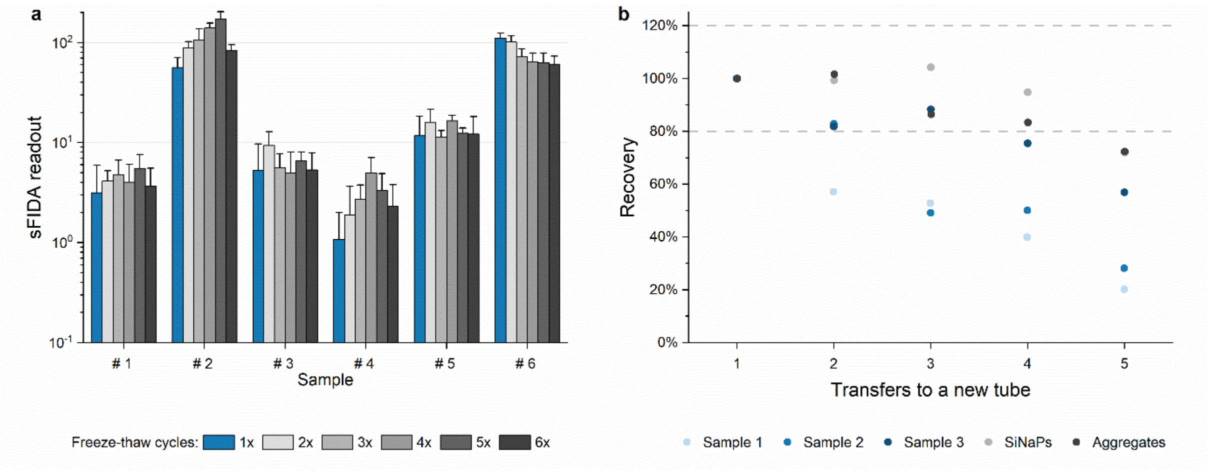

Supplementary Fig. 5 Evaluation of freeze-thaw cycles (a) and influence of tube transfers (b).

a Six patient plasma samples were thawed for 2 h at RT and then refrozen. The sFIDA readout for half of the samples remained unaffected (mean recovery 103%–129%), whereas additional freeze-thaw cycles caused an increase in the sFIDA readout for two samples and a decrease in the readout for one sample. Please, note the logarithmic scaling. Standard deviation is calculated across the four replicates. **b** After centrifugation, the supernatants of the plasma samples were transferred to a new tube (one transfer). One additional transfer reduced the sFIDA readout for the samples, whereas that of SiNaPs and recombinant aggregates remains constant for at least two additional transfers. Standard deviation was calculated across the four replicates. Please, note the logarithmic scale.

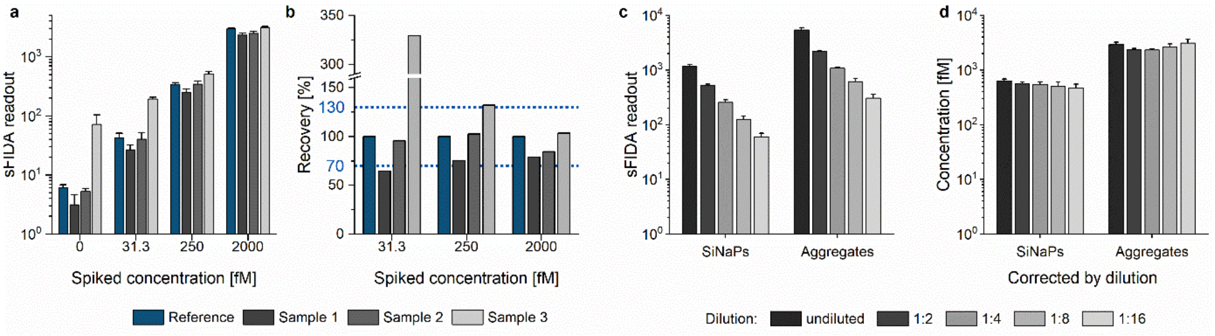

Supplementary Fig. 6 Recovery of SiNaPs in different matrices (a, b) and dilution of SiNaPs and aggregates spiked in plasma (c, d).

a Three samples with an initial low (sample 1), medium (sample 2) and high concentration (sample 3) of Aβ oligomers were spiked with three different concentrations of SiNaPs to examine whether the recovery of SiNaPs is dependent on the individual plasma matrix. **b** The obtained sFIDA readouts were initially normalized with the non-spiked sample and then compared to the readout of the same concentration of SiNaPs in the reference plasma sample. Samples 1 and 2 showed a mean recovery of 73% and 94%, respectively. For sample 3, the lowest concentration of 31.3 fM was beyond the acceptable range (dashed blue line), whereas medium and high concentrations of SiNaPs spiked in sample 3 showed a mean recovery of 118%. Mean recovery of all samples and concentrations (except sample 3 spiked with a low concentration) was 92%. **c** SiNaPs and recombinant $A\beta_{1-42}$ aggregates were spiked in plasma and diluted two-fold with TBS. **d** After calibration and correction for dilution, calculated stock concentrations for SiNaPs and aggregates differed on average by 15.9% and 8.9%, respectively. Standard deviations are calculated across the four replicates. Please, note the logarithmic scaling in a, c and d.

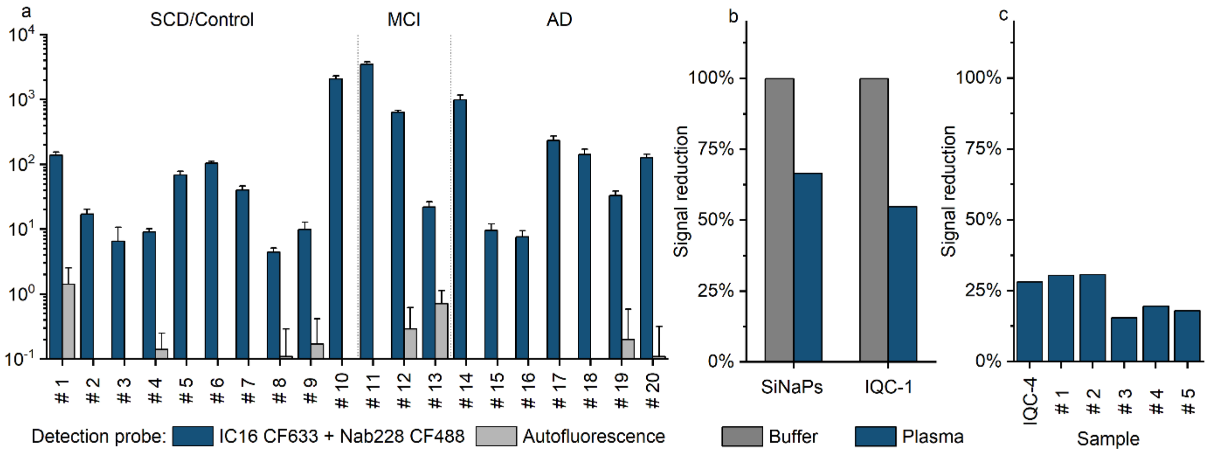

Supplementary Fig. 7 Autofluorescence of plasma samples

a Autofluorescence of 20 plasma samples was evaluated by incubation with the detection buffer without adding the capture antibody. Mean sFIDA readout was 0.61% compared to the signal with detection antibodies IC16 CF633 and Nab228 CF488 while all autofluorescence signals were below LOD. The experiment was carried out as part of the assay development, which is why there are slight deviations from the final protocol with regard to capture antibody (Nab228 at 2.5 µg/ml), blocking concentration (3% BSA) and washing steps after sample and detection antibody incubation (washing steps only with TBS). **b** To test unspecific binding of the analyte to the assay surface, sFIDA assay was performed with and without capture antibody with capture control refereing to the signal without capture antibody. Leaving of the capture antibody lead to a signal reduction of >99% for 8 pM SiNaPs and Aβ1−42 aggregates (IQC-1 with 18 ng/ml Aβ1−42 monomer concentration) spiked in low cross buffer while the same concentration spiked in a plasma samples resulted in a signal reduction of 33.5% and 54.7%, respectively. **c** Signal reduction for capture control for a lower concentration of Aβ1−42 aggregates (IQC-4 with 4.45 ng/ml Aβ1−42 monomer concentration) was at 28.1% while signal reduction in capture control for plasma samples ranged from 15.5% to 30.7% with a mean signal reduction of 22.8%, respectively. Standard deviation was calculated across the four replicates. Please, note the logarithmic scaling.

Supplementary Table 1 Mean Spearman coefficient of correlation r of the bootstrapping analysis of Aβ oligomers in plasma with demographics and biomarkers in CSF and plasma (*p*-value of Spearman distribution * 0.01 – 0.05, ** *p*-value 0.001 – 0.01)

		Control/Relatives /SCD	p-value	MCI/AD	p-value
	Age	−0.100	0.078	0.047	0.313
	MMSE	−0.053	0.242	0.011	0.451
**CSF**	$A\beta_{1-40}$	−0.037	0.314	0.219*	0.015
	$A\beta_{1-42}$	0.103	0.083	0.236**	0.005
	$A\beta_{1-42}/A\beta_{1-40}$	0.166*	0.013	0.196*	0.022
	tTau	−0.160*	0.015	0.023	0.410
	pTau	−0.203**	0.002	0.051	0.302
	Aβ Oligomers	0.186**	0.005	−0.217**	0.009
	Tau Oligomers	0.201**	0.002	−0.096	0.165
**Plasma**	$A\beta_{1-40}$	−0.067	0.205	0.158	0.111
	$A\beta_{1-42}$	−0.044	0.300	0.054	0.336
	$A\beta_{1-42}/A\beta_{1-40}$	0.034	0.337	−0.233*	0.031

Supplementary references

1 Herrmann, Y. *et al.* Nanoparticle standards for immuno-based quantitation of alpha-synuclein oligomers in diagnostics of Parkinson's disease and other synucleinopathies. *Clin Chim Acta* **466**, 152-159 (2017). https://doi.org:10.1016/j.cca.2017.01.010
