# Supplementary material for: Blood-based quantification of Aβ oligomers indicates impaired clearance from brain in ApoE ε4 positive subjects
Source: Commun Med (Lond). 2024 Dec 10;4:262. doi: 10.1038/s43856-024-00690-w (PMC11631981; doi:10.1038/s43856-024-00690-w)
Supplement: Supplementary file 3 — Description of Additional Supplementary File [file 43856_2024_690_MOESM3_ESM.pdf]

Description Of Additional Supplementary File

File name: Supplementary data

Description: Original data of Figures
