# Supplementary material for: Blood-based quantification of Aβ oligomers indicates impaired clearance from brain in ApoE ε4 positive subjects
Source: Commun Med (Lond). 2024 Dec 10;4:262. doi: 10.1038/s43856-024-00690-w (PMC11631981; doi:10.1038/s43856-024-00690-w)
Supplement: Supplementary file 5 — Reporting Summary [file 43856_2024_690_MOESM5_ESM.pdf]

nature portfolio

Corresponding author(s): Dieter Willbold

Last updated by author(s): Sep 6, 2024

Reporting Summary

Nature Portfolio wishes to improve the reproducibility of the work that we publish. This form provides structure for consistency and transparency in reporting. For further information on Nature Portfolio policies, see our Editorial Policies and the Editorial Policy Checklist.

Statistics

For all statistical analyses, confirm that the following items are present in the figure legend, table legend, main text, or Methods section.

n/a	Confirmed	
☐	☒	The exact sample size (*n*) for each experimental group/condition, given as a discrete number and unit of measurement
☐	☒	A statement on whether measurements were taken from distinct samples or whether the same sample was measured repeatedly
☐	☒	The statistical test(s) used AND whether they are one- or two-sided<br>*Only common tests should be described solely by name; describe more complex techniques in the Methods section.*
☐	☒	A description of all covariates tested
☐	☒	A description of any assumptions or corrections, such as tests of normality and adjustment for multiple comparisons
☐	☒	A full description of the statistical parameters including central tendency (e.g. means) or other basic estimates (e.g. regression coefficient) AND variation (e.g. standard deviation) or associated estimates of uncertainty (e.g. confidence intervals)
☐	☒	For null hypothesis testing, the test statistic (e.g. *F*, *t*, *r*) with confidence intervals, effect sizes, degrees of freedom and *P* value noted<br>*Give P values as exact values whenever suitable.*
☒	☐	For Bayesian analysis, information on the choice of priors and Markov chain Monte Carlo settings
☒	☐	For hierarchical and complex designs, identification of the appropriate level for tests and full reporting of outcomes
☐	☒	Estimates of effect sizes (e.g. Cohen's *d*, Pearson's *r*), indicating how they were calculated

Our web collection on statistics for biologists contains articles on many of the points above.

Software and code

Policy information about availability of computer code

Data collection	Leica Application Suite Advanced Fluorescende (LAS AF) 3.2.9653
Data analysis	MS Excel 2019<br>Origin Pro 2020<br>Python (included skikit learn library, version 1.0.2)<br>sFIDAta

For manuscripts utilizing custom algorithms or software that are central to the research but not yet described in published literature, software must be made available to editors and reviewers. We strongly encourage code deposition in a community repository (e.g. GitHub). See the Nature Portfolio guidelines for submitting code & software for further information.

Data

Policy information about availability of data

All manuscripts must include a data availability statement. This statement should provide the following information, where applicable:

- Accession codes, unique identifiers, or web links for publicly available datasets
- A description of any restrictions on data availability
- For clinical datasets or third party data, please ensure that the statement adheres to our policy

The authors confirm that the data supporting the findings of this study are availabe within the article and its supplementary materials or can be made available upon request.

Human research participants

Policy information about studies involving human research participants and Sex and Gender in Research.

Reporting on sex and gender	the terms "sex" and "gender" were not used in the manuscript. To compare the individual patient groups, the percentage of females within each group were reported. Analysis were based on the complete set of samples not considering sex.
Population characteristics	for population characteristics, refer to table 1 of the manuscript
Recruitment	retrospective analysis of biobanked samples; recruiting was described in Jessen 2018 (DOI: 10.1186/s13195-017-0314-2)
Ethics oversight	The project was approved by the ethical committee of the Charité Berlin (EA1/074/21 and EA4/066/17)

Note that full information on the approval of the study protocol must also be provided in the manuscript.

Field-specific reporting

Please select the one below that is the best fit for your research. If you are not sure, read the appropriate sections before making your selection.

☒ Life sciences ☐ Behavioural & social sciences ☐ Ecological, evolutionary & environmental sciences

For a reference copy of the document with all sections, see nature.com/documents/nr-reporting-summary-flat.pdf

Life sciences study design

All studies must disclose on these points even when the disclosure is negative.

Sample size	no sample size calculation since this was a retrospective analysis of biobanked samples
Data exclusions	- based on pre-clinical tests, all samples showing a visible redish color indicating haemolysis were excluded from analysis (n = 7)<br>- one sample needs to be excluded as no clinical data were available (diagnosis, CSF or plasma biomarkers)<br>- all samples of one out of seven experiments (in total 62 samples) were excluded from analysis because of a contamination of an assay component
Replication	data replication is described in the manuscript (cf intra-assay, inter-assay measurements)
Randomization	no randomization
Blinding	all samples were blinded at the time of measurement to the technicicans who measured and analyzed the data

Reporting for specific materials, systems and methods

We require information from authors about some types of materials, experimental systems and methods used in many studies. Here, indicate whether each material, system or method listed is relevant to your study. If you are not sure if a list item applies to your research, read the appropriate section before selecting a response.

Materials & experimental systems

n/a	Involved in the study
☐	☒ Antibodies
☒	☐ Eukaryotic cell lines
☒	☐ Palaeontology and archaeology
☒	☐ Animals and other organisms
☒	☐ Clinical data
☒	☐ Dual use research of concern

Methods

n/a	Involved in the study
☒	☐ ChIP-seq
☒	☐ Flow cytometry
☒	☐ MRI-based neuroimaging

Antibodies

Antibodies used

Bapineuzumab, proteogenix, article number: PX-TA1180, lot: 093021-A01
Nab228, Sigma Aldrich, article number: A8354, lot: 064M4760
IC16, Heinrich-Heine Universität Düsseldorf, lot: 220502

Validation

Bapineuzumab (information based on suppliers website): Bapineuzumab Biosimilar - Anti-APP Abeta mAb, on SDS-PAGE under reducing and non-reducing condition. The gel was stained overnight with Coomassie Blue. The purity of the antibody is greater than 95%. (https://www.proteogenix.science/product/bapineuzumab-biosimilar-anti-app-abeta-mab-research-grade/); additional information to the antibody: Miles 2013 (DOI: 10.1038/srep01302)

Nab228 (information based on suppliers website): Immunogen: synthetic peptide corresponding to amino acids 1-11 of human β-amyloid protein.; The antibody recognizes human β-amyloid peptide, full-length amyloid precursor protein (APP), soluble-APP (sAPPβ' and sAPPα), C99 cleavage form, and Aβ (1-40/42), but not soluble-APP form sAPPβ. (https://www.sigmaaldrich.com/DE/en/product/sigma/a8354)

IC16: Verwey 2013 (DOI: 10.3109/13506129.2013.797389); Antonios 2013 (DOI: 10.1186/2051-5960-1-56)
